# Availability of disaggregated greenhouse gas emissions from beef cattle production: A systematic review

**DOI:** 10.1016/j.eiar.2019.02.003

**Published:** 2019-05

**Authors:** John Lynch

**Affiliations:** Department of Physics, University of Oxford, UK

**Keywords:** Cattle, Beef, Greenhouse gas, Carbon dioxide equivalent, Methane, Nitrous oxide, AR, Assessment Report, CO_2_e, Carbon dioxide equivalent, GHG, Greenhouse gas, GTP, Global Temperature change Potential, GWP, Global Warming Potential, IPCC, Intergovernmental Panel on Climate Change, LCA, Life Cycle Assessment, RE, Radiative Efficiency, RF, Radiative Forcing

## Abstract

Agriculture is a significant source of anthropogenic greenhouse gas (GHG) emissions, and beef cattle are particularly emissions intensive. GHG emissions are typically expressed as a carbon dioxide equivalent (CO_2_e) ‘carbon footprint’ per unit output. The 100-year Global Warming Potential (GWP_100_) is the most commonly used CO_2_e metric, but others have also been proposed, and there is no universal reason to prefer GWP_100_ over alternative metrics. The weightings assigned to non-CO_2_ GHGs can differ significantly depending on the metric used, and relying upon a single metric can obscure important differences in the climate impacts of different GHGs. This loss of detail is especially relevant to beef production systems, as the majority of GHG emissions (as conventionally reported) are in the form of methane (CH_4_) and nitrous oxide (N_2_O), rather than CO_2_. This paper presents a systematic literature review of harmonised cradle to farm-gate beef carbon footprints from bottom-up studies on individual or representative systems, collecting the emissions data for each separate GHG, rather than a single CO_2_e value. Disaggregated GHG emissions could not be obtained for the majority of studies, highlighting the loss of information resulting from the standard reporting of total GWP_100_ CO_2_e alone. Where individual GHG compositions were available, significant variation was found for all gases. A comparison of grass fed and non-grass fed beef production systems was used to illustrate dynamics that are not sufficiently captured through a single CO_2_e footprint. Few clear trends emerged between the two dietary groups, but there was a non-significant indication that under GWP_100_ non-grass fed systems generally appear more emissions efficient, but under an alternative metric, the 100-year global temperature potential (GTP_100_), grass-fed beef had lower footprints. Despite recent focus on agricultural emissions, this review concludes there are insufficient data available to fully address important questions regarding the climate impacts of agricultural production, and calls for researchers to include separate GHG emissions in addition to aggregated CO_2_e footprints.

## Highlights

• Multi-gas footprints are typically converted to a total carbon dioxide equivalent.
• Different carbon dioxide equivalence metrics emphasize different climate behaviours.
• Reporting emissions of all gases as a combined total loses important information.
• Disaggregated data could not be retrieved from most (71%) full beef LCAs.
• Relative emissions intensity is highly dependent on metric choice.

## 1 Introduction

Greenhouse gas (GHG) emissions from livestock are a significant contributor to anthropogenic global warming (Reisinger and Clark, 2018). Population growth, urbanisation and economic development are expected to increase the demand for livestock products (Thornton, 2010), resulting in increased emissions (Garnett, 2009). Beef production is particularly emissions intensive, and beef is often highlighted as having one of the largest GHG footprints among common food products (Clune et al., 2017). However, different beef production systems show significant variation in their total emissions (de Vries and de Boer, 2010) and composition of individual GHGs, which determines their climate impact (Pierrehumbert and Eshel, 2015). It is therefore important that sufficiently detailed emissions data are available.

### 1.1 Greenhouse gas emissions associated with beef production

Beef production includes a number of processes that generate GHG emissions (Desjardins et al., 2012). Methane (CH_4_) from enteric fermentation, part of the digestive process of ruminant animals in which carbohydrates are broken down by microbial activity, is generally the largest emissions source from beef production (under the most commonly used metric – see below). Animal excreta generate further emissions, with a proportion of organic content lost as additional CH_4_, and nitrogen lost as nitrous oxide (N_2_O). Nitrogen inputs to agricultural soils, including (but not limited to) fertiliser application, result in further N_2_O emissions, while urea and lime application result in carbon dioxide (CO_2_) emissions. CO_2_ emissions are also generated by on-farm energy use, either in the form of electricity or fuel. Land-use and land-use change greenhouse gas fluxes can also result from beef production, with CO_2_ either emitted to or sequestered from the atmosphere depending upon changes in plant biomass and soil organic content.

The emissions described above cover typical on-farm (‘within farm gate’) GHGs. To give a complete account of the emissions that are generated as a result of beef production, i.e. a life-cycle assessment (LCA), the system boundaries must be expanded beyond this to include the impacts incurred in the production of farm inputs (‘pre-farm gate’). These include, for example, any agricultural and land use emissions from the production of feedstuffs grown elsewhere, and the energy used to manufacture fertilisers and other inputs. Including these emissions then covers the production process from initial inputs (‘cradle’) to the point at which finished animals leave the farm, often referred to as ‘cradle-to-gate’.

System boundaries can be expanded further still, including downstream (post-farm gate) emissions resulting from, for example, transport of animals, abattoir energy and resource use, refrigeration and cooking (for a complete ‘cradle-to-fork’ LCA). This study focuses on cradle-to-gate emissions, as a commonly used system boundary for agricultural production LCAs. The greenhouse gas emissions component of an LCA is often referred to as the ‘carbon footprint’ of a product (here, the term ‘GHG footprint’ is preferred).

### 1.2 Carbon dioxide equivalence metrics

GHG footprints are typically expressed as a CO_2_ equivalent (CO_2_e) that equates different GHGs to CO_2_. Emissions of non-CO_2_ gases are multiplied by metric values that describe the amount of CO_2_ that would result in an equivalent climate impact. For multi-gas footprints, the CO_2_e values of each gas can then be summed to give a single combined CO_2_e footprint. As these metrics are relative to CO_2_, CO_2_ emissions are added without conversion. However, there are multiple CO_2_ equivalence metrics that can be expressed over different timescales, resulting in significant variation in conversion factors for the same GHG (e.g. Table 1). Metric choice can thus have a large impact on agricultural GHG footprints, and especially those associated with ruminant livestock, due to the extent of non-CO_2_ emissions.

**Table 1:** Twenty- and 100-year Global Warming Potential (GWP) and Global Temperature Potential (GTP) values for biogenic methane (CH_4_) and nitrous oxide (N_2_O) (without carbon cycle feedbacks). From Myhre et al. (2013).

	GWP_20_	GWP_100_	GTP_20_	GTP_100_
CH_4_	84	28	67	4
N_2_O	264	265	277	234

Alternative metrics differ markedly due to the distinct physical properties of individual GHGs. GHGs differ in their atmospheric lifespan and radiative efficiency (RE), the amount by which they alter the Earth's energy balance (measured as the change in radiative energy balance per change in atmospheric concentration of a given GHG). CO_2_ has a relatively low RE, but can persist for millennia (Archer and Brovkin, 2008). CH_4_ has a greater RE, but an average atmospheric lifetime of only around 12.4 years, while N_2_O has an even larger RE, and a lifetime of approximately 121 years (Myhre et al., 2013). The net energy balance perturbation resulting from a given change in the concentration of a GHG (generally over a specified time period) is defined as its radiative forcing (RF), and the total RF from all climate pollutants ultimately leads to warming as the Earth system adjusts (Myhre et al., 2013). CO_2_ equivalence metrics typically collapse differences in both atmospheric lifespan and RE into a single value by modelling the RF (or alternative consequential impact such as temperature change) that would result from a specified emission scenario. The RF (or other output measure) for a given GHG is then scaled relative to the value for CO_2_ in the same reference scenario. Such approaches, however, can mask important dynamics (Pierrehumbert, 2014).

The most commonly used CO_2_e metric, the 100-year global warming potential (GWP_100_), is the integrated RF for 100 years following a one-off pulse emission of a gas, relative to CO_2_, and could be considered as representing “the total energy added to the climate system by a component in question relative to that added by CO_2_” (Myhre et al., 2013) over this period. Due to the differing atmospheric lifespans of different gases, this ‘addition of energy’ relative to CO_2_ is not temporally uniform, and so the effect of changing the time horizon depends on the GHG. For a short-lived GHG such as methane, the increased atmospheric concentrations and hence elevated RF that result from a pulse emission will dissipate after a few decades, and so increasing the time-horizon beyond this increases the CO_2_ reference denominator (as the emitted CO_2_ and hence its resultant radiative forcing persist) while the methane numerator is unchanged, thus reducing its CO_2_ equivalence value. For longer-lived GHGs such as nitrous oxide, the impact of emissions remains relatively uniform over time, and so their CO_2_ equivalence values are less sensitive to changing the time-horizon (for time-horizons shorter than the lifetime of the gas).

The most prominent alternative metric, the Global Temperature change Potential (GTP, also described in the most recent IPCC Assessment Report), is based on the modelled temperature impact of different gases relative to CO_2_ at a specified time following an emission pulse (GTP_100_ for the temperature response 100 years following an emission). Here the time refers to a specific endpoint, rather than integrating over an increasing period, and so GTP is even more sensitive to changing time horizon than GWP. As it relates to a final modelled impact, it can be slightly easier to intuit the meaning of GTP changing over time than GWP: if the same quantities of CO_2_ and methane are released into the atmosphere, after 20 years the methane will cause a temperature increase 67 times greater than the CO_2_, but after 100 years that same methane emission will be responsible for a temperature increase only 4 times that of the emission of CO_2_ (Table 1). The CO_2_ equivalence for a given GHG is thus only defined by the specific aspect of the climate response described by the chosen metric, at the specific time horizon used. If only a single combined CO_2_ equivalent footprint is provided, dynamics outside of these specifications cannot be inferred. For example, if we know that an activity has a total GTP_20_ footprint of 67 kg CO_2_e, it could be 1 kg of methane (0 kg CO_2_), in which case 100 years after this activity the temperature impact would decline to the equivalent of 4 kg CO_2_, or the footprint could be 67 kg CO_2_ (0 kg methane), which would, by definition, still have the impact of 67 kg CO_2_ after 100 years.

Neither metric is more physically accurate than the other, as they are both derived from the same atmospheric behaviours, but incorporate different aspects of the climate response. In going further towards modelling climate responses, rather than just the processes that eventually result in temperature change, GTP is subject to more uncertainty than GWP, but it has been argued that this is also an important element in anticipating the climate response, and hence does not necessarily represent a disadvantage (Allen et al., 2016). Other metrics have also been suggested, but this paper demonstrates the fixed time-horizon GWP and GTP variants above taken from the most recent IPCC report (Myhre et al., 2013), as they remain the most widely used. The 20- and 100-year variants have been suggested as revealing shorter- and longer-term impacts of different GHGs respectively (Ocko et al., 2017, but see discussion below).

While GWP_100_ CO_2_e has become the standard metric used in emissions reporting and climate policy, it has been criticised (e.g. O'Neill, 2000; Fuglestvedt et al., 2003; Shine, 2009; Pierrehumbert, 2014), and the IPCC assessment reports remain cautious not to suggest any one metric is inherently superior: “All choices of metric contain implicit value-related judgements such as type of effect considered and weighting of effects over time” (Myhre et al., 2013). Comparing multiple climate metrics for a single footprint has been proposed as a means of better incorporating the differences between GHGs and acknowledging the impact of metric choice on any conclusions drawn (Levasseur et al., 2016), and recently recommended in new global guidance on environmental life cycle impact assessment indicators from the United Nations Environmental Programme and Society of Environmental Toxicology and Chemistry (Jolliet et al., 2018). Metric comparisons are only possible if GHG footprints are provided as separate emissions of individual gases: as noted above, it is not possible to work backwards or infer these effects from a single aggregated CO_2_e footprint.

Given that significant amounts of methane are emitted by cattle production, aggregated CO_2_e footprints for the activity will vary greatly depending on the metric used. As well as changing the apparent climate impacts of beef production relative to other activities, there may be important implications for the appraisal of different types of beef production. On-going debate surrounds the relative emissions efficiency of cattle fed diets of either predominantly grass or higher energy feeds (Capper, 2012). Higher energy feeds (e.g. grains or soy) result in reduced CH_4_ emissions, as they are more digestible and energy-dense (Knapp et al., 2014), and a number of recent reviews have illustrated, based on comparisons of total GWP_100_ CO_2_e footprints, that grass-fed cattle are generally less emissions efficient (de Vries et al., 2015; Clark and Tilman, 2017; Gerssen-Gondelach et al., 2017). However, if these lower GWP_100_ footprints are a result of lower animal CH_4_ but come at the expense of higher CO_2_ and N_2_O emissions incurred in producing feeds, this conclusion may only hold under metrics that value CH_4_ relatively highly. This adds important nuance to the types of system we should prioritise for climate sustainability, and links with wider concerns such as avoiding the consumption of human-edible foodstuffs by ruminant livestock (Eisler et al., 2014).

The complexities in cattle emissions and their resulting climate impacts make it essential that GHG footprints are available as separate emissions of individual gases. Despite this, there is a general focus on GWP_100_ as a reporting metric that has resulted in many footprinting papers (and reviews thereof) only publishing a single aggregated CO_2_e value. This paper presents a systematic review of bottom-up beef footprint studies, the primary purpose of which is simply to establish how GHG footprints are reported, and how readily available disaggregated emissions data are in the current literature. The consequences of alternative metric choices are explored, and illustrated with a simple example comparing grass fed and non-grass fed production systems.

## 2 Methods

### 2.1 Systematic literature search

Systematic review design and reporting were undertaken following the PRISMA checklist (Moher et al., 2009). The search string ‘(beef OR cow OR cattle) AND (emissions OR greenhouse OR GHG) AND (LCA OR “life cycle”)’ was searched for in the Sciencedirect, Pubmed and Web of Science online indexing and database services in March 2018. All studies found through these databases were screened for relevance based on title. Relevant titles were then screened by abstract, and the full text was then reviewed.

At the full paper inspection stage, a number of criteria had to be met to standardise systems included. Beef LCAs that were linked with dairy production (either from culled dairy cows or calves transferred to beef cattle systems) were excluded in order to standardise system types and avoid different dairy-beef co-product allocations (Rice et al., 2017). Only bottom-up studies of either real farms or detailed representative systems based on regional/national averages were included; footprints based on proposed/emerging mitigations or top-down approaches, including input-output LCA and global modelling, were excluded. System boundaries had to cover complete cradle to farm gate emissions. Emissions had to be expressed (or expressible) per unit of finished beef. Finally, to fulfil the main objective of this paper, individual emissions of CH_4_, N_2_O and CO_2_ needed to be retrievable, either reported in quantities of individual gases, or relative proportions of a final CO_2_e footprint. If a study was excluded only due to insufficient GHG disaggregation, the corresponding author was emailed to request this extra GHG breakdown, with two months to receive responses before final analyses were undertaken.

### 2.2 Data extraction

Relevant data were collated into a database for those studies that met the inclusion criteria. Paper details (lead author, year of publication and manuscript title) and a short description of the beef production system were included for each footprint. Where sufficient dietary information was available, systems were classified by predominant post-weaning diet, defined as either grass fed or non-grass fed depending on whether at least 50% (by dry matter) of their diet was grass-based (including grazing, silage, or hay); as opposed to typical non-grass feeds of grains, maize silage and soy. This diet categorisation is an extension of the concentrate or roughage classification used by de Vries et al. (2015), with ‘grass-fed’ distinct from ‘roughage’ due to the exclusion of maize silage.

The continent and country of each system was recorded, defined by endpoint if production spanned multiple countries. Due to the small number of data-points for many regions and sub-groups, these data were not used in analyses. The sample size (no. farms) of each footprint was also recorded, with *n* = 0 denoting that the study was based on a simulated and/or representative system. Where papers included multiple footprints over time, either the most recent or only those based on farm management data (c.f. more speculative scenarios) were recorded.

Emission details were then recorded. A preliminary assessment identified finished cattle liveweight as the most common output measure, so this was adopted as the standard output unit. Conversions were undertaken for studies that reported carcass weights. If the paper reported its own dressing percentage this was used to convert back to liveweight, otherwise region-specific standards from Opio et al. (2013, Table B20) were used.

For the emissions themselves, the CO_2_e conversion factors used in the paper were first recorded, either based on the IPCC Assessment Report quoted or the paper's reporting of individual CH_4_ and N_2_O GWPs. Where different gases were expressed only as CO_2_e or a proportion of a total CO_2_e footprint they were converted back to quantities of individual gases. Harmonised CO_2_e footprints were generated using the IPCC Fifth Assessment Report (AR5) 20- and 100-year GWP and GTP conversion factors (Myhre et al., 2013). Where studies explicitly included land-use change emissions or sequestrations these were also recorded, however as they were not presented for most footprints and were not possible to standardise, these data were not included in analyses.

### 2.3 Analyses

Simple summary statistics were used to demonstrate the range in emissions for individual gases and aggregated total CO_2_e emissions derived using 20- and 100-year variants of GWP and GTP.

Relationships between individual gas emissions were explored using the Kendall rank correlation coefficient. Tied rankings were adjusted for using Kendall's tau-b. False discovery rate (FDR) correction was applied.

The impact of harmonising footprints to AR5 GWP_100_ was investigated by comparing harmonised CO_2_e values with those reported in the original papers using a paired Wilcoxon signed rank test. Kendall's rank correlation coefficient was used to assess relationships between all individual footprints, comparing correlations between GWP_100_ against GTP_100_, and GTP_20_ against GTP_100_. Mann-Whitney *U* tests were used to compare the two dietary groups, comparing all three individual gases and total GWP_100_ and GTP_100_ footprints.

Analyses were performed using R (R Core Team, 2018) and the ‘Kendall’ package (McLeod, 2015).

No weightings were applied in averaging or comparing across studies, as there was no adequate means of assigning universal weighting factors. This risks pseudo-replication, particularly where multiple footprints from individual studies rely on some shared data, but was deemed necessary to cover a sufficient range of systems and not omit useful comparisons. As a result of this limitation, and further concerns over the aggregation of independent LCA studies (discussed below), the results presented here should be considered an illustration of the types of issues arising from the lack of disaggregated GHG data, rather than a reliable demonstration of what having these data can resolve.

## 3 Results

### 3.1 Beef GHG footprint literature

**Fig. 1:**
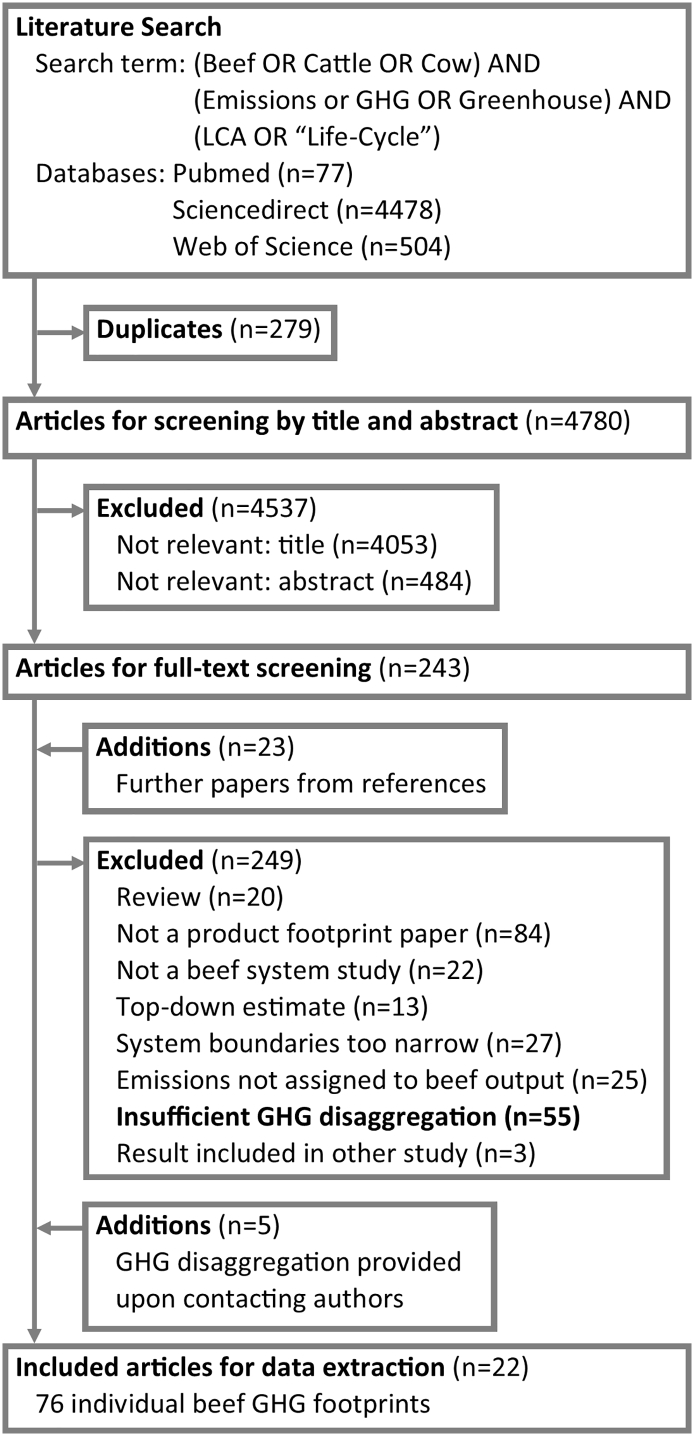
Systematic literature review flow chat, n = number of papers.

The systematic review resulted in a total of 76 individual beef GHG footprints from 22 peer-reviewed papers (Fig. 1). Of most importance to this review, a large number (*n* = 55) of studies were excluded as GHG emissions were not reported with sufficient disaggregation, and so the necessary data could not be obtained from the CO_2_e footprint(s) as published.

Papers generally used an attributional LCA approach (although this was rarely explicitly stated). In two included studies (Buratti et al., 2017; Parajuli et al., 2018), a system expansion approach was used to remove manure emissions where they displaced fertiliser application, but these emissions were re-included in footprints to standardise with other papers in this review.

All papers included in this study and their emissions data are available in a linked spreadsheet.

### 3.2 Disaggregated greenhouse gas emissions from beef production

**Fig. 2:**
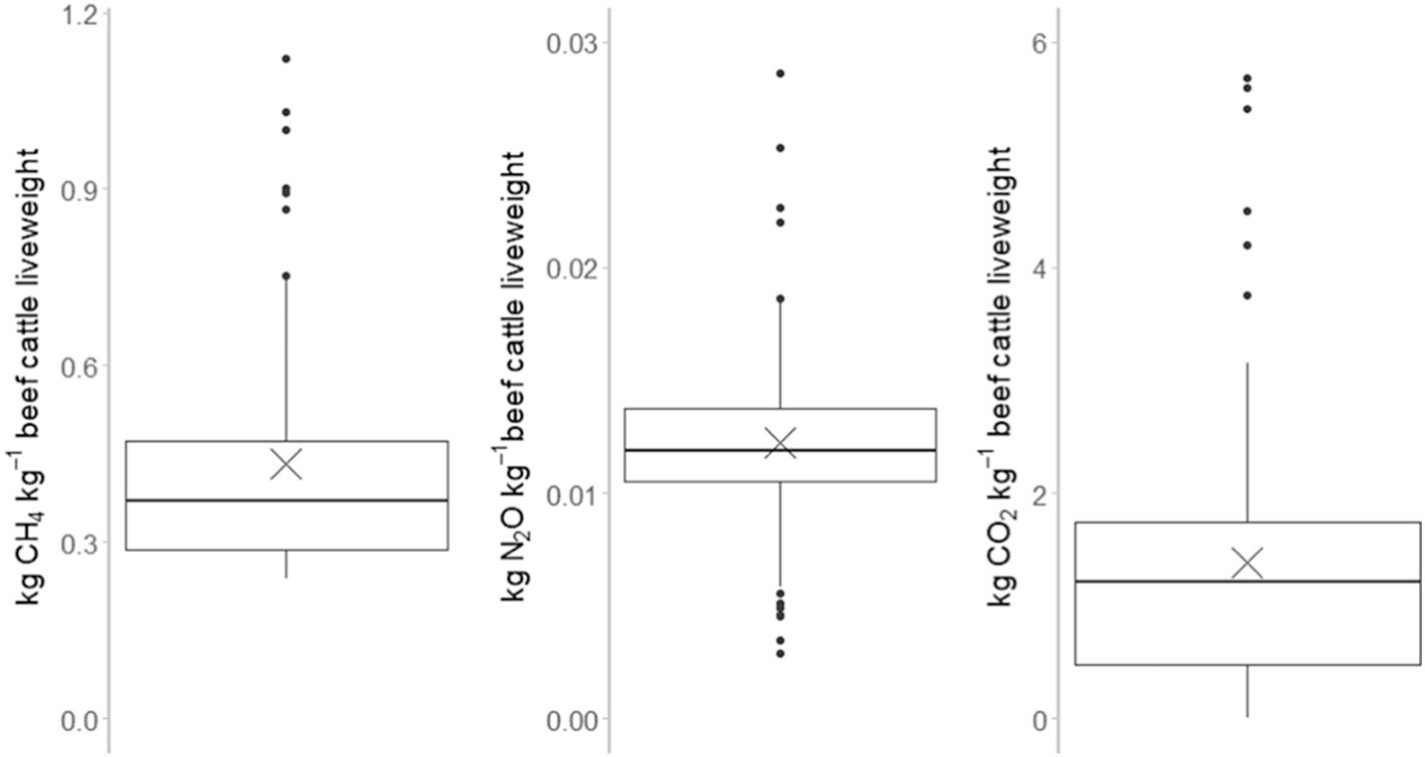
Emissions of CH_4_, N_2_O and CO_2_ per unit kg beef finished liveweight.

The beef GHG footprints collected displayed a large range for each gas (Fig. 2). CH_4_ emissions ranged from 0.24 to 1.12 kg kg^−1^ liveweight ($\bar{x}$=0.43, SD = 0.20), N_2_O from 0.0029 to 0.0286 kg kg^−1^ liveweight ($\bar{x}$=0.012, SD = 0.0046) and CO_2_ from 0 to 5.68 kg kg^−1^ liveweight ($\bar{x}$=1.39, SD = 1.27).

**Fig. 3:**
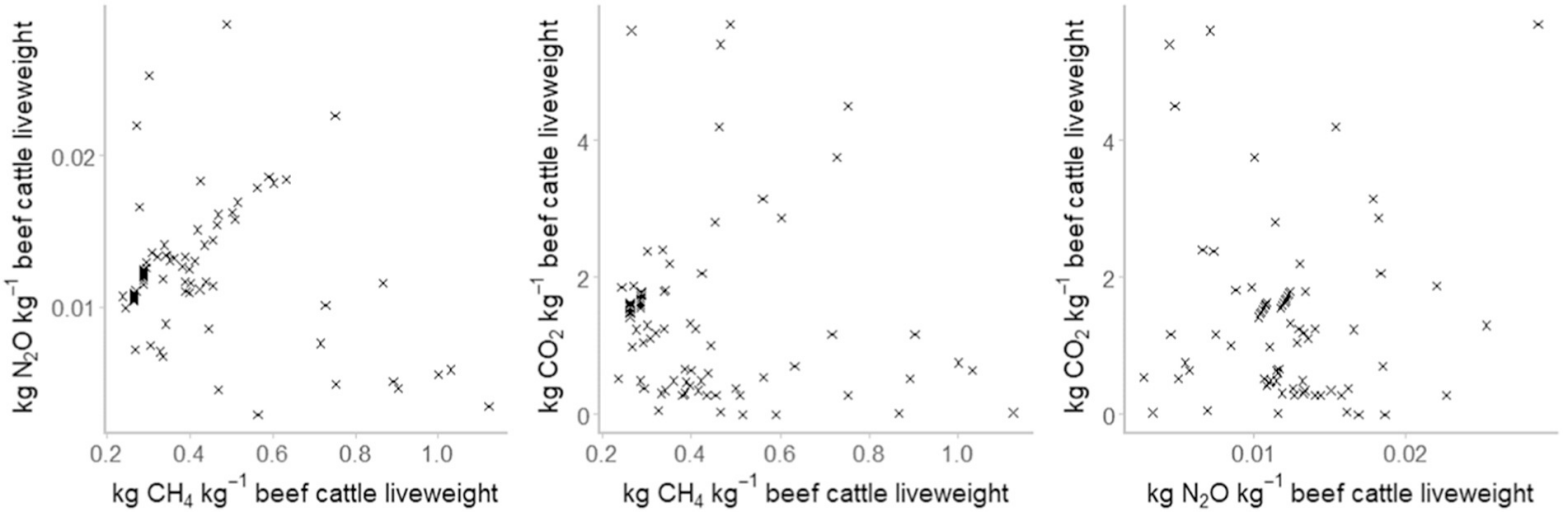
Relationships between individual greenhouse gas emissions for each beef footprint. Correlations were non-significant in each case (adj. p = 0.15, 0.09 and 0.23 for relationships between CH_4_ and N_2_O, CH_4_ and CO_2_, and N_2_O and CO_2_ respectively).

Relationships between all individual gases were tested using Kendall's tau rank correlation (Fig. 3), finding no clear trend between CH_4_ and N_2_O (r_τ_ = 0.13, *p* = 0.10, FDR adj. *p* = 0.15) or N_2_O and CO_2_ (r_τ_ = −0.09, *p* = 0.23, FDR adj. *p* = 0.23), but limited evidence for a weak negative association between emissions of CH_4_ and CO_2_ (r_τ_ = −0.17 *p* = 0.03, FDR adj. *p* = 0.09).

### 3.3 Carbon dioxide equivalence metrics

**Fig. 4:**
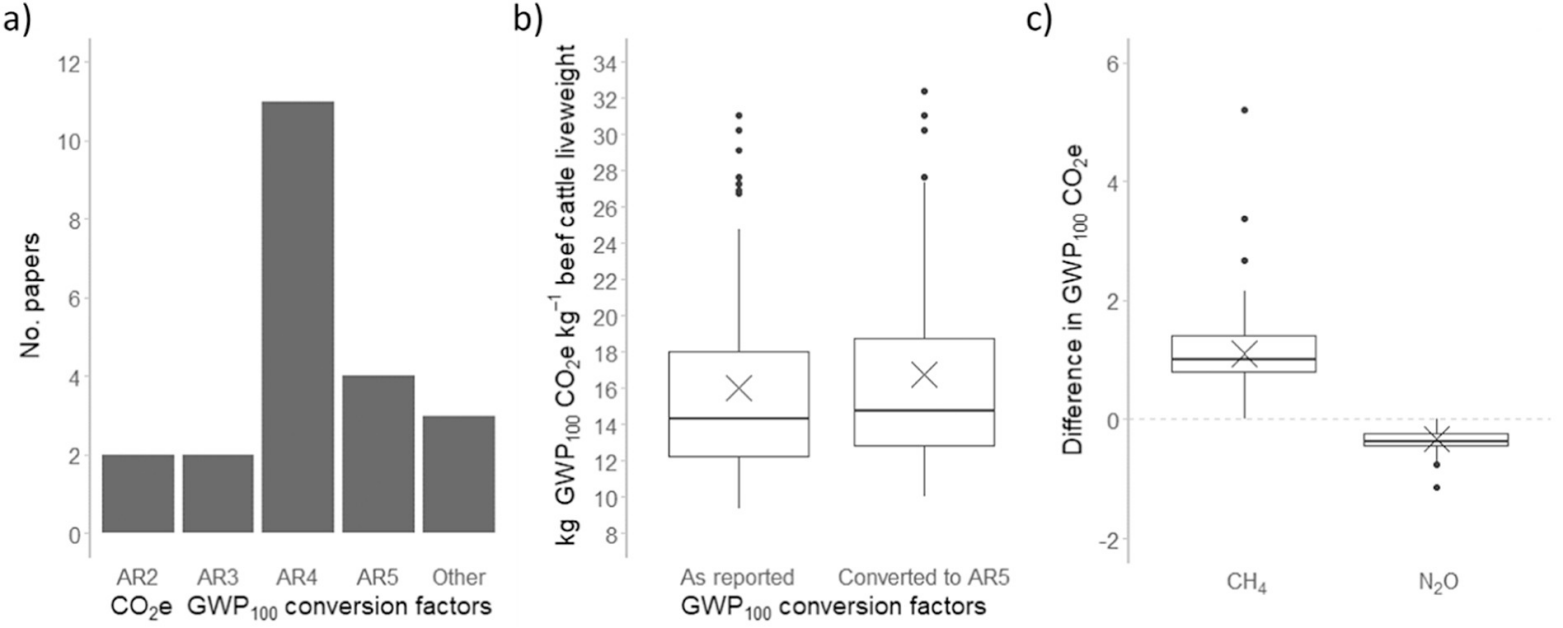
The usage and impact of different GWP_100_ CO_2_e conversion factors, showing (a) the different conversion factors used (by which IPCC Assessment Report, AR, they were from), (b) the overall impact of harmonising to AR5 GWP_100_ conversion factors, and (c) the difference in the paper's own and AR5 harmonised CH_4_ and N_2_O emissions reported as GWP_100_ CO_2_e.

The majority of papers reported emissions using IPCC fourth assessment report (AR4) GWP_100_ conversion factors, but studies also reported footprints using the AR2, AR3, AR5 and other GWP_100_ conversion factors (Fig. 4a). It was not always clear where the non-IPCC conversion factors were from, but in general they seemed to be alternative lower values for CH_4_ that offset the CO_2_ resulting from CH_4_ oxidation (following Muñoz et al., 2013) before this was incorporated by increasing the non-biogenic CH_4_ conversions factors in AR5 (Myhre et al., 2013). The net effect of harmonising to AR5 GWP_100_ conversion factors was a significant increase in total CO_2_e footprint compared to the paper's original reported value (Fig. 4b, paper's original footprints: median = 14.30, $\bar{x}$ = 16.00; AR5 harmonised footprints: median = 14.76, $\bar{x}$ = 16.76; *Z* = 1.63, *p* < 0.001), as the CO_2_e reductions resulting from the new, lower N_2_O conversion factor were more than overcome by the increases due to the higher CH_4_ conversion factor (Fig. 4c).

**Fig. 5:**
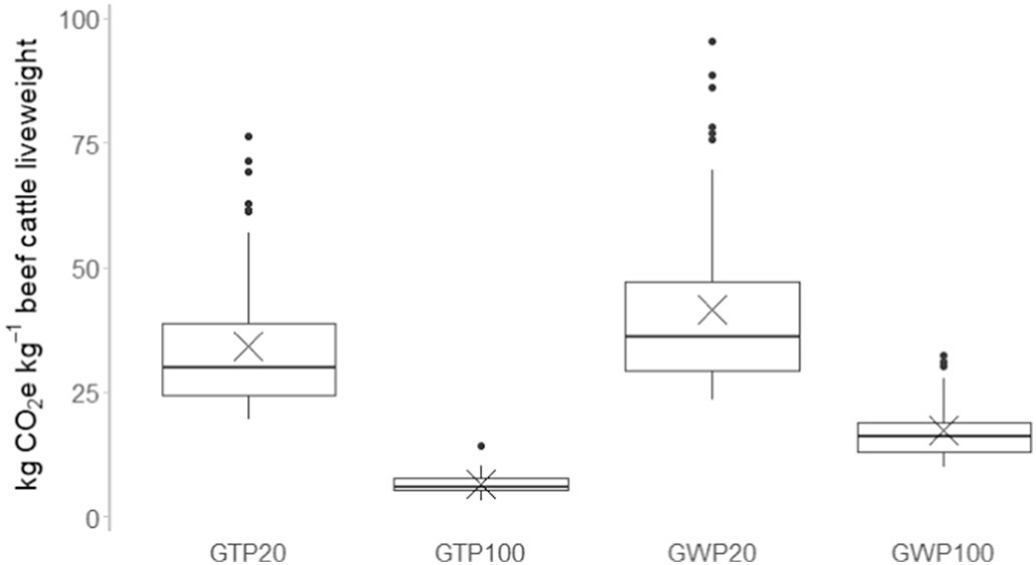
CO_2_e footprints of beef cattle production under alternative metrics.

Due to the large amount of methane in footprints, AR5 harmonised metric choice strongly influenced the total CO_2_e footprint (Fig. 5). The average GWP_100_ footprint for all studies in the review ranged from 10.02 to 32.37 kg CO_2_e kg^−1^ liveweight ($\bar{x}$=16.76, SD = 5.56), while the harmonised GTP_100_ footprints were between 3.01 and 14.34 kg CO_2_e kg^−1^ liveweight ($\bar{x}$=5.99, SD = 1.71). The 20-year variants showed much greater footprint values and much larger ranges: 23.34 to 95.24 kg CO_2_e kg^−1^ liveweight ($\bar{x}$=41.42, SD = 16.31) for GWP_20_ and 19.43 to 76.20 ($\bar{x}$=34.22, SD = 13.18) for GTP_20_.

**Fig. 6:**
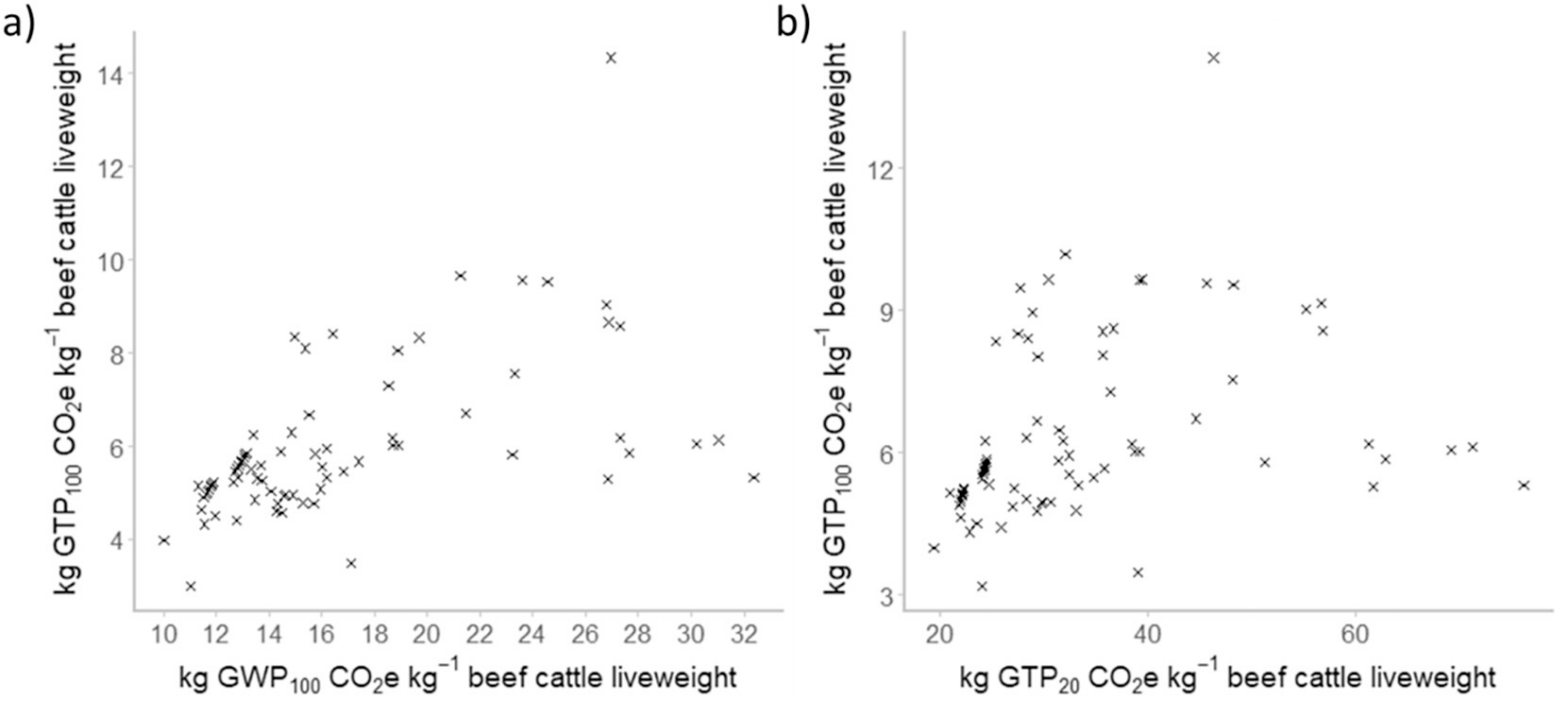
Relationships between a) GWP_100_ and GTP_100_ (r_τ_ = 0.48, p < 0.001) and b) GTP_20_ and GTP_100_ (r_τ_ = 0.39, p < 0.001) CO_2_e footprints per kg beef cattle liveweight.

There was a significant correlation between GWP_100_ and GTP_100_ footprints, with beef systems ranked highly under one metric likely to be similarly placed in the alternative, but there was also considerable variation around this relationship reflecting how the two metrics can differ based on an alternative balances of gases (Fig. 6a, r_τ_ = 0.48, *p* < 0.001), and highlighting that a lower total CO_2_e for a given metric does not necessarily correspond to lower emissions of all gases. An alternative but related way of considering this is provided by comparing GTP_20_ and GTP_100_ (Fig. 6b, r_τ_ = 0.39, *p* < 0.001): the temperature impact after 20 years is strongly correlated with the temperature impact after 100 years, but is clearly not a direct predictor.

### 3.4 Emissions efficiency of different production systems

**Fig. 7:**
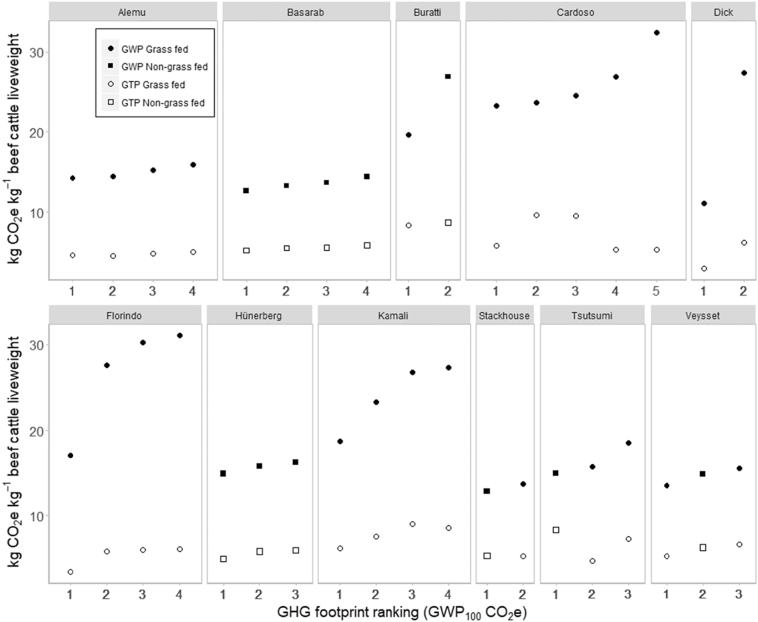
Differences in GWP_100_ and GTP_100_ CO_2_e for studies comparing multiple systems. Boxes represent the following individual studies, labelled by lead author: Alemu et al. (2017), Basarab et al. (2012), Buratti et al. (2017), Cardoso et al. (2016), Dick et al. (2015), Florindo et al. (2017), Hünerberg et al. (2014), Kamali et al. (2016), Stackhouse-Lawson et al. (2012), Tsutsumi et al. (2018), Veysset et al. (2010).

To illustrate some of the implications of these dynamics for the apparent emissions efficiency of different systems, GWP_100_ and GTP_100_ footprints were compared for a number of studies containing multiple footprints, and hence guaranteed methodological standardisation (Fig. 7). As would be expected, GTP_100_ resulted in universally lower footprints than GWP_100_. In many cases the relative rankings of each system were the same under both metrics, but the proportional improvement between systems could still show significant differences; for example in the study of Buratti et al. (2017), using GTP_100_ the non-grass fed system appears to be marginally more emissions efficient, but its apparent increase in efficiency is much more pronounced under GWP_100_. In some cases metric choice determined the relative ranking of different systems. For example in Tsutsumi et al. (2018) the ‘conventional’ system had the lowest GWP_100_, footprint, as the cattle were fed a large proportion of concentrates, resulting in lower CH_4_ emissions. These concentrates, however, were associated with significant CO_2_ emissions from their growth in and transport from the USA. The two alternative systems feeding farm-grown roughage had much lower CO_2_ emissions at the expense of more CH_4_. As GTP_100_ values CH_4_ much less strongly than GWP_100_, the apparently superior emissions efficiency of the conventional over grass-fed systems was reversed.

**Fig. 8:**
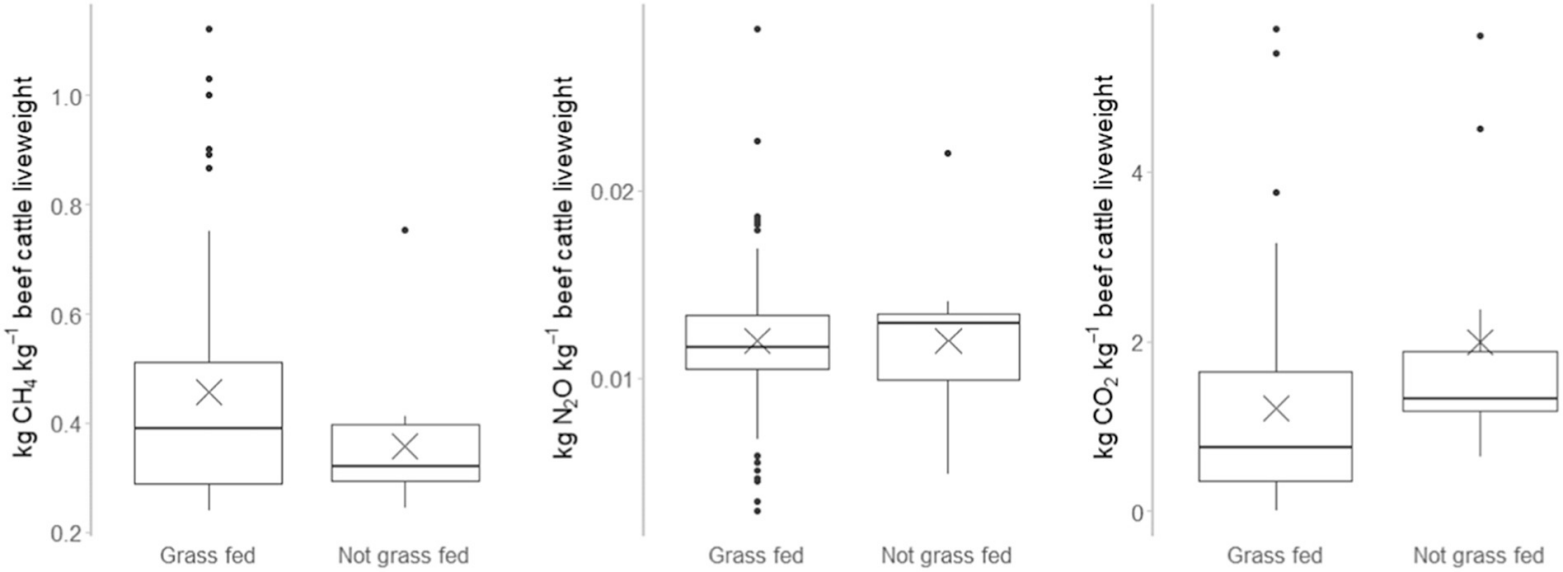
Emissions of CH_4_, N_2_O and CO_2_ per kg beef cattle liveweight for different feed types.

Comparing emissions by predominant feed-type for all systems where this classification was available (Fig. 8), a large range in emissions was again observed for both grass fed (CH_4_: $\bar{x}$=0.46, SD = 0.22; N_2_O: $\bar{x}$=0.012, SD = 0.005; CO_2_: $\bar{x}$=1.22, SD = 1.19) and non-grass fed systems (CH_4_: $\bar{x}$=0.36, SD = 0.13; N_2_O: $\bar{x}$=0.012, SD = 0.004; CO_2_: $\bar{x}$=1.99, SD = 1.45), indicating that emissions are largely driven by wider differences. There was no clear association between CH_4_ and N_2_O emissions and feed type (CH_4_: U = 484, Z = 1.47, *p* = 0.14, FDR adj. *p* = 0.21; N_2_O: U = 349, Z = −0.51, *p* = 0.61, FDR adj. *p* = 0.61), but there was some evidence that CO_2_ emissions were lower in grass fed than non-grass fed systems (CO_2_: U = 484, Z = 1.47, *p* = 0.02, FDR adj. *p* = 0.07).

**Fig. 9:**
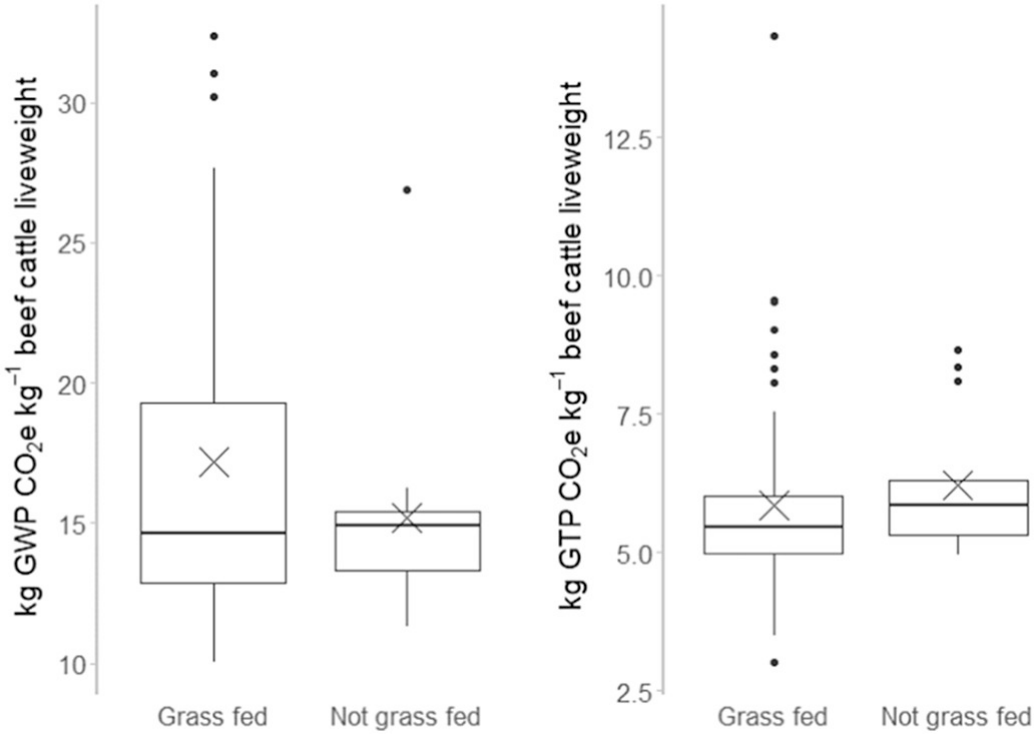
Grass fed and non-grass fed GWP_100_ and GTP_100_ CO_2_e footprints per kg beef cattle liveweight.

Total CO_2_e footprints for grass fed and non-grass fed systems were compared to establish whether, across all-studies, feed-type altered the balance of different GHG emissions such that relative performance changed according to metric choice (Fig. 9). The most emissions efficient grass-fed systems were optimal under either metric, but there was an overall trend for grass-fed systems to have larger CO_2_e footprints under GWP_100_, while they tended to have lower footprints than non-grass fed systems under GTP_100_. However, the differences in CO_2_e footprint between feed types were not significant under either metric (GWP100: *Z* = −1.31, *p* = 0.19; GTP100: *Z* = 1.63, *p* = 0.10).

## 4 Discussion

### 4.1 Importance of GHG disaggregation

Reporting GHG emissions footprints as only the total GWP_100_ CO_2_e loses important information on their composition of different GHGs, which greatly limits our ability to make meaningful comparisons or investigate the climate impacts of different products or production systems.

The resulting lack of clear climate inference can be illustrated by considering beef GHG footprints in relation to crude oil combustion (as a reference activity that primarily emits CO_2_). Taking just a single footprint of 1.59 kg CO_2_, 0.43 kg CH_4_ and 0.012 kg N_2_O, the average emissions across all systems this review, the GWP_100_ footprint of 16.81 kg CO_2_e would suggest that producing 1 kg of cattle liveweight is equivalent to the combustion of approximately 6 l of crude oil^1^. These same emissions could also be considered equivalent to the CO_2_ emitted from burning from 2 to 15 l of crude oil using either the GTP_100_ or GWP_20_ CO_2_e footprints, respectively, or anywhere between under alternative time-horizons. All of these values are a technically accurate description of the climate response to these emissions, representing different concepts of ‘carbon dioxide equivalence’ for their given timescales. The most meaningful or useful approach will depend on the specific questions posed or climate policy ambitions. No single metric that treats short- and long-lived GHGs in the same way can fully capture their different climate dynamics. Nor can we work backwards to infer these effects from a single aggregated CO_2_e footprint.

Many have questioned the utility of GWP_100_ CO_2_e footprints as a climate metric (see introduction), and as GWP_100_ is not necessarily related to either climate impacts or policy goals, relying on this metric may result in misleading or incomplete conclusions (Allen et al., 2016; Cherubini et al., 2016). Including alternative metrics and/or comparing multiple time-horizons has been recommended as a means for LCA studies to consider the implications of different choices and provide greater transparency (Cherubini et al., 2016; Levasseur et al., 2016; Jolliet et al., 2018). Despite this, it was only possible to derive separate emissions data from a relatively small proportion (29%) of published cradle-to-grave beef footprints, and only one study in this review, Picasso et al. (2014), considered an alternative metric (GTP_100_) in addition to GWP_100_.

The 20- and 100-year variants of GWP and GTP demonstrated here illustrate part of a wider debate around GHG metric choice and the most appropriate means of describing the climate impacts of different gases (or activities that emit them). It has been argued that using a 100-year time-horizon to indicate ‘long-term’ warming results in significant undervaluation of the impacts of CO_2_ relative to other gases, as its atmospheric lifespan extends well beyond 100 years (Pierrehumbert, 2014). Conversely, if metrics are based on too distant a time-horizon we may minimise long-term impacts but overshoot near-term climate goals. An alternative dynamic use of GTP, where the time horizon is determined by a specified target year (Shine et al., 2007), as demonstrated for beef footprints in Persson et al. (2015), shows one potential means of better linking metrics to policy goals. A modified use of GWP, GWP*, that relates a change in rate of emissions of short-lived gases (i.e. CH_4_) to cumulative total emissions of long-lived gases (N_2_O and CO_2_) has been suggested as a more useful means of equating their climate impacts (Allen et al., 2016; Allen et al., 2018). Carbon dioxide equivalence metrics can also be dispensed with altogether, using individual GHG emissions from agricultural production in climate models (Pierrehumbert and Eshel, 2015). Comparing a greater range of metrics or climate modelling were beyond the scope of this review, but highlight the same fundamental principle, as disaggregated emissions would be required to explore any of these alternatives.

As well as significantly changing the apparent emissions intensity of beef production in relation to other climate polluting activities, the relative emissions efficiency of different types of cattle system is also influenced by metric choice. It has been demonstrated that for New Zealand dairy production, metrics such as GTP_100_ that value CH_4_ relatively less highly favour low-input systems, as the increased animal CH_4_ emissions that can result from lower-intensity production are more than offset by lower emissions of longer-lived gases (Reisinger and Ledgard, 2013; Reisinger et al., 2017). This trend was demonstrated in some instances here, but there were few clear trends between different gases overall. While Reisinger et al. (2017) found reasonable homogeneity and broadly consistent ranking among their sample of Waikato dairy farms, the geographic and system type variation in this review meant that few overarching patterns were observed. Although some dynamics are universal and can be assumed (e.g. longer time horizons will always give lower CO_2_e values for CH_4_), the highly system specific emissions of individual GHGs (and relationships between them) suggest we cannot reliably infer climate impacts without disaggregated data. In the context of the simple comparison of grass and non-grass fed systems presented here, there were indications that the apparently optimum form of production is dependent on metric, but the nature of current emissions reporting leaves us ill-equipped to fully interrogate the topic, despite significant public interest and a number of studies exploring the issue.

Even if limiting assessment to differences in GWP_100_ CO_2_e footprints (or any other single metric), without the emissions of individual gases it becomes impossible to standardise footprints published at different times as the CO_2_e conversion factors are revised. McAuliffe et al. (2018) provides a rare acknowledgement of this, demonstrating in a recent beef cattle production study that using the newer conversion factors significantly increased the apparent emissions intensity in some systems due to the greater GWP_100_ value for CH_4_, as observed for the harmonised AR5 footprints in this review. Metric values are updated between different assessment reports as atmospheric conditions change and further climate research is incorporated. Recent research has indicated an upwards revision of the radiative efficiency of methane, which would result in increased CO_2_e conversion factors (Etminan et al., 2016). Hence it is likely that current footprinting studies will again become irreversibly outdated following the next IPCC assessment report unless their emissions are available in disaggregated form. In addition, it has been argued that climate‑carbon feedbacks should potentially be included in standard CO_2_e metrics (Gasser et al., 2017), and incorporated in climate change indicators in environmental impact assessments (Jolliet et al., 2018). Updating past studies to include these feedbacks would also require disaggregated emissions data.

### 4.2 Beef footprint reviews and sustainable food systems

A growing body of research attempts to describe the impacts of current and projected diets (e.g Tilman and Clark, 2014), and suggest what changes might be necessary to keep the required agricultural production within sustainable limits (e.g. Springmann et al., 2018). Extending the dynamics described above, moving beyond GWP_100_ would provide a more detailed and meaningful appraisal of the climate impacts of agriculture and more clearly relate emissions to given time-frames and goals. Considerations around GHG metrics are also especially prominent in light of the Paris Agreement, given its focus on temperature targets and (currently) unspecified choice of metric (Fuglestvedt et al., 2018).

Reviews such as this must consider the limitations of averaging or comparing individual footprints across multiple studies, given the very large ranges in results, differences in LCA methodologies, and difficulties in confirming the representativeness of a given study. While these difficulties are acknowledged, the exploratory nature of this study, strict inclusion criteria and harmonisation are suggested as justifying the approaches presented here, which are also in keeping with the wider literature (e.g. reviews of food product GHG footprints such as Clune et al., 2017, and food system sustainability studies as above). This highlights the somewhat contradictory positions of agricultural environmental impact assessment and food system sustainability research where, for example, reviews of only beef footprints can be deemed too broad to combine individual results (de Vries et al., 2015), but large-scale global dietary models aggregate and compare results from hugely disparate food product footprints (Tilman and Clark, 2014). Agricultural product footprinting studies need to be adequately standardised so that we can reliably compare ‘apples to kangaroos’ (Hawkins et al., 2016), yet the difficulty in standardising even across a single product illustrates the challenges in achieving this. In addition to the primary message to report disaggregated GHG emissions, this paper reiterates calls for wider improvements and standardisation in agricultural LCAs, including the need for more transparent and location-specific databases and emission factors, and consistent methods and system boundaries (Notarnicola et al., 2017; Adewale et al., 2018). As demonstrated here, for example, even in otherwise complete cradle to farm gate beef LCAs, it was not possible to standardise the treatment of land-use emissions and/or sequestrations, highlighting a particular difficulty in the context of ruminant livestock environmental impact assessments.

### 4.3 Beef GHG emissions in wider context

Although GHG emissions were the focus of this study, the wider impacts and broader context of beef production must also be acknowledged (McClelland et al., 2018). Additional negative environmental consequences beyond GHG emissions are also associated with beef systems, including risks of acidification and eutrophication of local water bodies and land degradation (de Vries et al., 2015). At the same time, benefits beyond meat production may also be conferred, including ecosystem service provision and rural employment (Smith et al., 2013).

Standardising all emissions per kg of generic meat output is also a potentially reductive approach. Meat differs in a large range of attributes of potential consumer importance (Henchion et al., 2014). These other attributes can depend upon system type, with evidence that grass-fed beef may be nutritionally superior (Daley et al., 2010; McAfee et al., 2011), for example. Different meat attributes and a range of potential benefits or disbenefits of beef production must therefore be considered in order to fully appraise the relative value of different systems and the net impact of beef production. Assessing these multifaceted concerns can be complex, but provides important insight into the potential for agricultural sustainable intensification and how we might achieve healthy and sustainable diets. Improving our assessment of the climate impacts of different food products and production systems will provide an essential contribution to these topics.

## 5 Conclusions

Greenhouse gas emissions should not overshadow the other impacts of beef systems, whether wider negative externalities, or potential benefits beyond food provision. However, beef production is frequently highlighted as an especially emissions intensive activity, and so it is important to interrogate this topic specifically. This study suggests that relevant data are not as widespread or robust as they may first appear. Very high levels of beef consumption are climatically unsustainable, regardless of carbon dioxide equivalence metric (Pierrehumbert and Eshel, 2015). However, there are important details that we cannot reliably ascertain from the current literature. The standard reporting of GHG emissions as only a total GWP_100_ CO_2_e footprint results in a significant loss of information, and the main aim of this review is to draw attention to this and encourage researchers and practitioners to publish any emissions in a disaggregated form. Without this data, the inferred climate impacts of a given GHG footprint are not clear, and limiting reporting to total GWP_100_ CO_2_e can have significant implications for the apparent emissions efficiency of, for example, different types of beef production system, or the relative climate impact of beef production compared to other GHG-emitting activities. Even if using only a single climate metric, results cannot be standardised over time unless emissions of individual gases are known. Adding this data, even if just as a supplementary note, could immediately benefit research into the climate impacts of agricultural activity, and should be a straightforward addition, as individual studies or LCA databases must, at some point, have dealt with disaggregated emissions data before converting and summing to a total CO_2_e footprint. A greater awareness of debates surrounding carbon dioxide equivalence metrics and more consideration given to metric choice or the incorporation of climate modelling approaches can significantly improve the assessment of agricultural emissions.

## Footnotes

1 Assumes 0.0733 kg CO_2_ emitted per MJ crude oil combustion (IPCC, 2006), and 0.026 l per MJ (US EIA, 2018). Crude oil combustion also emits other GHGs, but can be considered a predominantly CO_2_ emitting activity for this comparison, as the ratio of CO_2_: CH_4_: N_2_O emissions is 73,300: 3: 0.6 (IPCC, 2006).
